# Identification of a locus for autosomal dominant high myopia on chromosome 5p13.3-p15.1 in a Chinese family

**Published:** 2010-10-12

**Authors:** Jun-Hua Ma, Shu-Hong Shen, Guo-Wei Zhang, Dong-Sheng Zhao, Chao Xu, Chun-Ming Pan, He Jiang, Zhi-Quan Wang, Huai-Dong Song

**Affiliations:** 1 Ruijin Hospital, State Key Laboratory of Medical Genomics, Molecular Medicine Center, Shanghai Institute of Endocrinology, Shanghai Jiao Tong University School of Medicine, Shanghai, China; 2 Department of Hematology/Oncology, Shanghai Children's Medical Center, School of Medicine, Shanghai Jiao Tong University, Shanghai, China; 3 School of Basic Medical Sciences, Hangzhou Normal University, Hangzhou, Zhejiang, China; 4 Department of Ophthalmology, Xinhua Hospital Affiliated to Shanghai Jiao Tong University School of Medicine, Shanghai, China

## Abstract

**Purpose:**

Myopia and its extreme form, high myopia, are common vision disorders worldwide, especially in Asia. Identifying genetic markers is a useful step toward understanding the genetic basis of high myopia, particularly in the Chinese population, where it is highly prevalent. This study was conducted to provide evidence of linkage for autosomal dominant high myopia to a locus on chromosome 5p13.3-p15.1 in a large Chinese family.

**Methods:**

After clinical evaluation, genomic DNA from 29 members of this family was genotyped. A genome-wide screen was then performed using 382 markers with an average inter-marker distance of 10 cM, and two-point linkage was analyzed using the MLINK program. Mutation analysis of the candidate genes was performed using direct sequencing.

**Results:**

Linkage to the known autosomal dominant high myopia loci was excluded. The genome-wide screening identified a maximum two-point LOD score of 3.71 at θ=0.00 with the microsatellite marker D5S502. Fine mapping and haplotype analysis defined a critical region of 11.69 cM between D5S2096 and D5S1986 on chromosome 5p13.3-p15.1. Sequence analysis of the candidate genes inside the linked region did not identify any causative mutations.

**Conclusions:**

A genetic locus was mapped to chromosome 5p13.3-p15.1 in a large Chinese family with autosomal dominant high myopia.

## Introduction

Myopia, the most common eye disease worldwide, is also the leading cause of visual impairment [1]. The prevalence of myopia has been increasing in recent decades, especially in East Asian areas such as Japan, Singapore, and China [2-4]. The Chinese appear to be more susceptible to myopia than other populations. The prevalence of myopia in primary school children aged 5 to 16 years of Hong Kong is 36.71% [5]. In adult persons more than 40 years old, Chinese residing in Singapore have a prevalence of myopia as high as 38.7% while the prevalence observed in European-derived populations in United States and Australia are 26.2% and 17% respectively [3,6,7]. High myopia, which is defined as a refractive error equal to or below −6.00 diopters (D), is also more prevalent in Chinese than Caucasian populations [3,8]. Individuals with high myopia have a greater chance of subsequently developing serious complications, including glaucoma, retinal detachment, and choroidal neovascularization, which if not treated early and appropriately, may lead to permanent visual impairment or blindness [9-11].

Genetic and environmental factors both contribute to the development of myopia. Environmental factors such as work at close range and prolonged reading are suggested to be involved in the progression of myopia [1,12,13] and a body of evidence supports the idea that heredity plays a central role in the etiology of myopia. Twin studies have reported a high degree of heritability for myopia, with monozygotic twins being more highly correlated than dizygotic twins [14,15]. In addition, the children of parents with myopia tend to have myopia more frequently than children of parents without myopia [16].

The inheritance of high myopia is equivocal. It may be inherited as an autosomal dominant, autosomal recessive, or X-linked recessive trait. Genetic mapping studies have identified at least 18 chromosomal regions suspected of harboring a myopia gene. X-linked recessive inheritance myopia has been mapped on Xq28 (MYP1) and Xq23–25 (MYP13) [17,18]. In addition, Yang et al. [19] found that the locus at 14q22.1-q24.2 (MYP18) was responsible for high myopia in a consanguineous Chinese family in an autosomal recessive pattern. Some research groups focusing on autosomal dominant high myopia have identified suggestive linkages on chromosome 18p11.31 (MYP2) [20], 12q21–23 (MYP3) [21], 7q36 (MYP4) [22], 17q21–22 (MYP5) [23], 4q22-q27 (MYP11) [24], 2q37.1 (MYP12) [25], 10q21.1 (MYP15) [26], and 5p15 (MYP16) [27]. Furthermore, certain loci have also been implicated in common myopia: 22q12 (MYP6) [28], 11p13 (MYP7), 3q26 (MYP8), 4q12 (MYP9), 8p23 (MYP10) [29], 1p36 (MYP14) [30], and 7p15 (MYP17) [31]. Identifying the genetic markers for myopia would be a useful step toward understanding the molecular defects that lead to the pathophysiology of myopia.

In this study, we recruited a four-generational Chinese family with autosomal dominant high myopia. Through genome-wide screening and linkage analysis, we mapped the disease to a locus on chromosome 5p13.3-p15.1.

## Methods

### Family and clinical evaluation

**Figure 1:**
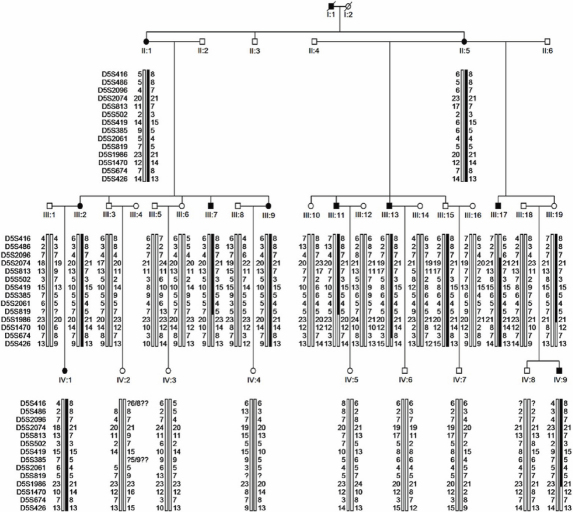
Pedigree and haplotype diagram of the family. Circles and squares denote females and males, respectively; blackened symbols denote affected individuals; a diagonal line through a symbol means that the individual is deceased. Haplotypes were constructed on the basis of the minimum number of recombinations between these markers. Solid bar: the chromosome assumed to carry the inherited disease allele; open bar: normal haplotypes. Only essential members are shown; nonparticipating family members were excluded. For individuals IV:2, only one set of parental-allele information was available; therefore, the genotype information was indeterminate (denoted by question marks) for markers D5S416 and D5S385. Individual III:17 was recombinant for the telomeric marker D5S2096. Individuals III:7 and III:11 were recombinant for the centromeric marker D5S1986.

A large family with autosomal dominant high myopia was identified in Zhejiang province, China. This family contained 11 affected individuals over four generations. Participating in this study were 29 individuals (aged 11 to 80 years): 10 affected and 19 unaffected (Figure 1). The study conformed to the guidelines involving human research as stated in the Declaration of Helsinki. Informed consent was obtained from every subject after an explanation of the nature, procedures and possible consequences of the study. This family was chosen based on the presence of numerous male and female family members and successful multiple generations with high myopia, suggesting an autosomal dominant mode of inheritance. Individuals with a spherical refractive error equal to or lower than −6.00 D, axial length longer than 26 mm in at least one eye and a history of myopia onset before 12 years of age were considered affected. Ophthalmology examination was performed for all of the members. No participant had any known ocular disease or insult that could predispose to myopia, such as a history of retinopathy of premature or neonatal problems, or a known genetic disease or connective tissue disorder associated with myopia, such as Stickler or Marfan syndrome. The results of the ophthalmic examinations are summarized in Table 1.

**Table 1:** Clinical information of individuals in the family.

**Subject**	**Gender**	**Myopia phenotype**	**Age at onset (years)**	**Age at exam (years)**	**Refractive Error OD**	**Refractive Error OS**	**Axial Length (OD;OS [mm])**
II:1	F	A	7	80	−11.00DS −4.00DC×100	−16.00DS −2.00DC×105	29.24; 30.10
II:5	F	A	8	79	−10.50DS −0. 50DC×90	−12.00DS −2.00DC×100	27.92; 27.57
III:1	M	NA		45	+1.00DS +1.00DC×180	+1.00DS +1.00DC×180	NP
III:2	F	A	11	43	−2.50DS −0.50DC×40	−11.50DS −1.50DC×150	22.62; 26.80
III:3	M	NA		49	+0.50DS sph	+0.50DS +0.50DC×180	23.04; 23.01
III:5	M	NA		55	+0.75DC×180	+0.75DC×180	24.11; 24.20
III:6	F	NA		48	+1.00DS +0.75DC×180	+1.00DS +1.00DC×180	22.82; 22.59
III:7	M	A	6	41	−24.00DS −2.00DC×40	−26.00DS −2.00DC×120	31.42; 31.18
III:8	M	NA		46	−4.50DS −0.50DC×90	−3.50DS −0.50DC×90	25.40; 25.51
III:9	F	A	11	43	+0.50DS −1.50DC×40	−15.50DS −1.50DC×140	22.92; 28.81
III:10	F	NA		37	+0.50DS +0.50DC×20	+1.00DS sph	22.61; 22.03
III:11	M	A	6	40	−16.00DS −1.00DC×60	+0.50DS −2.50DC×160	31.21; 25.60
III:12	F	NA		36	+0.50DS sph	+0.50DS sph	22.72; 22.49
III:13	M	A	5	45	−10.00DS sph	−9.00DS −1.00DC×120	29.40; 29.32
III:14	F	NA		36	+1.00DS −0.50DC×180	+1.00DS sph	23.41; 23.02
III:15	M	NA		41	+0.50DS sph	+0.50DS sph	23.42; 23.57
III:16	F	NA		39	+0.75DS sph	+0.75DS sph	23.02; 23.11
III:17	M	A	5	46	−17.00DS sph	−15.00DS sph	29.54; 31.56
III:18	M	NA		58	+1.00DS +1.00DC×180	+1.00DS +0.50DC×180	23.78; 23.81
III:19	F	NA		58	+1.00DS +0.75DC×180	+1.00DS +0.50DC×180	22.45; 22.37
IV:1	F	A	6	15	−13.00DS −3.00DC×10	−10.00DS −5.00DC×165	28.27; 27.73
IV:2	F	NA		25	−0.75DS sph	−0.50DS sph	23.30; 23.02
IV:3	F	NA		28	−5.50DS sph	−5.50DS sph	25.41; 25.50
IV:4	F	NA		17	−4.25DS −1.00DC×170	−5.00DS −1.00DC×170	25.55; 25.72
IV:5	F	NA		11	+0.50DS sph	+0.50DS sph	22.31; 22.49
IV:6	M	NA		16	−1.00DS sph	−1.00DS sph	24.41; 24.29
IV:7	M	NA		13	+0.50DS sph	+0.50DS sph	23.95; 23.86
IV:8	M	NA		30	+1.50DC×90	+1.75DC×90	22.67; 23.93
IV:9	M	A	4	32	−6.50DS −2.25DC×30	−8.00DS −2.00DC×170	28.03; 27.81

In the table, “A” indicates affected; “NA” indicates not affected; “M” indicates male; “F” indicates female; “OD” indicates right eye; “OS” indicates left eye; “NP” indicates not preformed; “sph” indicates sphere; and “mm” indicates millimeters.

### Genotyping and linkage analysis

Genomic DNA was isolated from peripheral blood leucocytes by the standard proteinase K digestion and phenol-chloroform extraction. The genome-wide screen was conducted on ABI 3700 sequencer by using PRISM Linkage Mapping Set MD-10 (Applied Biosystems, Inc., Foster City, CA) that have 382 highly polymorphic fluorescent markers with an average spacing of 10 cM. The markers were ampliﬁed by polymerase chain reaction (PCR) under the following conditions: 50 ng genomic DNA, 2 pmol each primer, 0.2 μl dNTP (10 mM each), 1 μl 10× buffer, 0.6 μl MgCl_2_ (25 mM) and 0.4 U HotStar Taq DNA polymerase (Qiagen, Santa Clarita, CA) in a ﬁnal volume of 10 μl. Six to eight primer pairs were multiplexed in the ampliﬁcation reaction. Samples were incubated in a PTC-225 DNA Engine Tetrad (MJ Research, Waltham, MA) for 15 min at 95 °C to predenature, followed by 35 cycles of 30 s at 94 °C, 40 s at 55 °C, 40 s at 72 °C, and a ﬁnal extension at 72 °C for 10 min. Ampliﬁcation products were appropriately pooled into prescribed panels, diluted, and denatured for 5 min at 95 °C, then incubated on ice for 2 min. Subsequently, the products were run in an automated DNA sequencer (ABI Prism 3700; Applied Biosystems).

Data were analyzed using GeneScan 3.7NT and Genotyper 3.7NT software (Perkin Elmer, Foster City, CA). Two-point LOD scores were calculated using the MLINK program from the Linkage software package (version 5.2). For fine mapping, additional microsatellite markers spanning the chromosome 5p region were selected from the genetic map of the Marshfield Center for Medical Genetics (Marshfield, WI). The myopia in the family was analyzed as an autosomal dominant trait with 90% penetrance and with a disease-gene allele frequency of 0.01. Recombination frequencies were assumed to be equal between males and females. Haplotype analysis was performed with Cyrillic software (version 2.0) and confirmed by inspection.

### Positional candidate gene mutation screening

The identiﬁed genes located in the linkage region were proposed as candidate genes on the basis of their functional information. Mutations of these genes were screened by direct sequencing. Using the soft Primer Express 2.0 (Perkin Elmer), primers were designed to amplify each exon including exon-intron boundaries regions of the candidate genes from genomic DNA (the sequences of all primers used in this study are summarized in Table 2). Screening for mutations was initially performed in two affected and two unaffected individuals. The PCRs were performed using Taq DNA polymerase and the products were sequenced directly with a dye-terminator cycle-sequencing system by ABI Prism 3700 DNA sequencer after puriﬁed by exonuclease I (Epicenter, Madison, WI) and shrimp alkaline phosphatase (USB, Cleveland, OH). The resulting sequences were compared with the corresponding wild-type sequences using Autoassembler software (version 2.0; Perkin Elmer). When a sequence variant was detected, the exon was ampliﬁed from the genomic DNA extracted from the other individuals to determine whether the base variant was speciﬁc to the patients. The NCBI SNP database was also referenced to determine whether the sequence variant was a polymorphism.

**Table 2:** Primers designed for mutation screening.

Exon	Primer Sequence (F,R)	Melting Temperature (°C)	Product Size (bp)
*CDH6*			
1	F:CAGACGGAGTCATACAAGTTCTGAG	58	824
	R: GCCCTTTGGTAATTGACCAGC	59	
2	F:GCACATGCCTTCCATTTAGC	57	770
	R: GGTTGTGGGTGTTTCAACTGG	59	
3	F: CTCCCAAACTCTGTTCCAGTTC	57	775
	R: TCTCTTTCAACCTCCCACTCC	57	
4	F: CCAAAGTTCTCGACTTCCTCAG	57	369
	R: GTGTTTGGTGGATGTATGCAAG	57	
5	F: ATCTATCTCCCCTGTGTGGTTG	57	521
	R: TTCCTGAGTGTATGCCATGTTG	57	
6	F: AAGAAGAACAGGCCACCATTAG	56	583
	R: GGTTTTGCCATGTTGGTCTC	57	
7	F: CATTTTTCAGGGCTGTGTGG	58	592
	R: CATCTTTTCTCAAGTGCAGGC	56	
8	F: GGTGATCTTCAAAGTCATGCAAC	57	538
	R: GAAACATTACTGCAAACCACTCC	57	
9	F: GTCTAAAGGGAATCGGCAATC	56	397
	R: TGGAATCAGCCTCAGTCTTTG	57	
10	F: TACTGATATCTCGTGGGTAGAGGC	58	552
	R: GCAAAGTGGTGAATGTATGTGG	57	
11	F: GCCAGTGGCTCAAACTTTACC	57	573
	R: CAGGCTGTATGCCTTAATGGG	58	
12	F: ATCATGGATGGAGGCAAGTG	57	756
	R: AACGGGTAGAACAGAGAAGCC	58	
*CDH10*			
1	F: CTATCAGCAGAACCTTTCTCTCCG	60	562
	R: CAAACATTTATCTCCTCCCTCTCC	58	
2	F: CACAACAGAAGGCGTGATTCC	59	603
	R: TGCTTCCTCACTGAACTCAATAGC	59	
3	F: TTTACCAAGCAAAGACAGGAGC	57	672
	R: CTCATGGTAGCAAATCAAAGAGG	57	
4	F: TTGCTCCTCCTTCTGGTACTGTG	59	626
	R: TTCATGTTCGGTAAAGCAGTCC	58	
5	F: GTGGTATTGCTAGGAAAGGGTAAC	56	600
	R: GGATCATAGGTCTTCCTGTCTCTG	57	
6	F: AAAAGCCCCGGAAGTTCCTAG	60	611
	R: CAGGTTTCCTGTCTCAATCAACC	59	
7	F: TGTAACTGGGTGGGAGCATATC	58	467
	R: AGTAGAGACAGGGTTTCACCATG	56	
8	F: TCAGTGATATGTGTGGGTTTGC	57	521
	R: CGGCCTGTTAATCTGTTTCATG	58	
9	F: CACTTCATACCCACAAGATGCC	58	577
	R: GCATTCGTCTCTCATCTCTCTAGC	58	
10	F: TAAAGGGTATGATCCAAAAGACAC	56	587
	R: ATCTCCAGCCGTTCTAATCTTATC	56	
11	F: GTAAGCACACACGCACAGATG	56	997
	R: TTTCCAAGCTCCTACACATGC	57	
12-1	F: TGCTAAACCCTTCAGCGTCTC	58	779
	R: AAATTGTGCTGACTGGCAGG	57	
12-2	F: TCCATTGCTGAATCTCTGAGTTC	57	889
	R: CATAGCATATCAAGACTCGCTGG	58	
*CDH12*			
1	F: CAGGTGACAGTTCTCTGATATGC	55	679
	R: ATCCCAATCAAAAACGGAAG	55	
2	F: CAATAGTGATAATCAGGTGTGAGG	58	398
	R: TTGTTGTGTTTATGTCAACTCCTTG	57	
3	F: TGCTGATTAGGATGTGGGC	56	403
	R: TCCAGTCTGGGTGAAAGAGTG	56	
4	F: AGCGTTCTTGCTAATCAGGTC	55	367
	R: GAAATTCAGTGCCATGCAGTC	57	
5	F: GGCAGATATAATGAGCGTTGTG	56	537
	R: CCTTCCTATCAAGCGGTTGTC	57	
6	F: TGGCACATCCTTTTAATGGTG	57	421
	R: TGTTGAAGAGGTCTCATTGTATCTC	55	
7	F: CGAGTCCAGCAGATAAGAGTCATG	59	233
	R: GGCTGGTGATAATGTTGCCTC	58	
8	F: ATATTTCTCATTGTGGCATGGC	58	511
	R: GCTTCCTAAAGACTAAGTGTCTGG	57	
9	F: GCCCAGTTAAAATTCTAGAGCAGC	59	560
	R: CACGGAGTATCAGTACCCCAAC	57	
10	F: TCACCATTTCTGCCACATTC	56	414
	R: CATCATGCAGTTTTGGACAGAG	57	
11	F: TTTCCCCTTGAGCATACTGAC	56	399
	R: GAAAAACATCTCAGCAGGGAC	55	
12	F: TGTAGAAGCAATAACTGACCGG	56	384
	R: TCATCTGTGCGGTATCACCTC	57	
13	F: CCTCTTTGAAACTGATGACCG	56	366
	R: ACAATGCAAGCAACCTGCC	58	
14	F: CTATTGAGCAGATACCAACTTGAAG	55	385
	R: AAAAAAAGGAAGAGAGAGCGAG	55	
15-1	F: CATTCACGAAACTCAGCCAC	55	521
	R: GATAGTCATAGTCCTGGTCGGC	56	
15-2	F: CTGCCCCACCATACGATTC	57	494
	R: ATAGGCCTGAGCTTGTCTCAG	55	
15-3	F: TCTGCCAACAAGAGATACATCC	57	632
	R: ATTTGAGCCCTGAGGCCTC	58	
*PDZD2*			
1-1	F: CTCTTCCTCTCCCAGGTGTGA	58	410
	R: AGGTGCAGCACGGCATTG	60	
1-2	F: AGCCTGAACATGAACACAGGC	59	776
	R: TGGTGCTCCAGTTGAAGATGTG	60	
2	F: AAGCTCAATACCTCAGTCCATCG	58	720
	R: CAGCTTGTATTTCCCGCATG	58	
3	F: CCAGTTGCCACTAGCCACATC	59	615
	R: TGTGCTCAGAGGTGTGTTCAATT	58	
4	F: AACAATCGCATCCCCAACTG	59	587
	R: TGCGCCATTGCACTCCAG	61	
5	F: CCTGGCCTTTGAGGTAACCTT	58	425
	R: TGGTGTCTTTTCCCATCATGGT	60	
6	F: GGAATTGGCCAATGCAAGG	60	671
	R: TTGCTCCAAAAGCTGCAACTC	59	
7	F: GCCTGACACTAATGGCTTCAGC	60	630
	R: CTATGAGACGCCATCGTCTCC	58	
8	F: TTTATTAGAGCCGGGGTTTCAC	58	639
	R: TGCAATGGCTCATGCCTATAATC	60	
9	F: TTAACTCCAGGCCTGGTTAGGG	60	592
	R: CCAGCCCTAAGGTAAGAATGGAC	59	
10 and 11	F: GTGACATTCCTGGGAGCTAAGCTA	60	816
	R: AGAGCTCCATGCTACTGGTAACTTC	58	
12	F: GCCATGGGCTCATTCTATTAACAG	60	529
	R: GGGAAGCCAAGATCTAGAGTTCTC	58	
13	F: GCATAATGACCTTTGCCCACTT	59	575
	R: CTTACCTCCCACCAGACAAGTTTC	59	
14	F: GGAGGATGGTCATAATCCTTGG	58	691
	R: CTATCTCAAGCCTTCTCTGCCTG	58	
15	F: CTGGGATCATCATGCAGGTAGTAG	59	584
	R: TAAAGCCCCAAGTCCTGACTCAC	60	
16	F: GTTGTCAATTTAGCCGTTCTGAG	57	579
	R: AGTAGAGATGGGGTTTCACCATGT	59	
17-1	F: AGCCATGTTGCCTAGGCCTT	60	583
	R: CCATGCCCTCTGGGATACTC	58	
17-2	F: GACAGAGAAGGGGACTGCATT	57	725
	R: CCAGCCATCATCCTTTCCC	59	
18	F: TGGATCTGCTGGCTCCTAGTC	58	430
	R: TCTCAAACTCCTGACCTCAAGTG	57	
19-1	F: GCTGGTCTCGAACTCCTGACC	60	657
	R: AGGTGCATGGATTCCTGTCATT	59	
19-2	F: ACAATACCAGGAGGGTGGCTG	60	820
	R: CTTGTGCAAAGACACACGGG	59	
19-3	F: GACAGCACCTCCCTATCAGGC	60	800
	R: CATGATGGGCCTCCTAGCG	60	
19-4	F: GCATTAATGCAGCTGCCAGTC	59	705
	R: GCGAGACAAATGTCCTGATGC	59	
19-5	F: TGACAGAAACACCACAGCTGC	58	721
	R: GTATTCTGCCTTTCTAATGGCTG	56	
19-6	F: AATCAGGCTCTATCGCCAGGT	59	630
	R: ATCCGCTGCTTCACAGAGAA	57	
19-7	F: TATATAGTGTAAAGCCGCTGCTGG	59	779
	R: TGGGAACTCTGCATTATCTTTGC	59	
20	F: ATTCATAGCAGTTCCCCTTGCC	60	520
	R: CTGAAGCTGGCTAGCAGCAC	58	
21	F: AGAACCTTTAGGGCCTGTGG	57	638
	R: AAGGTGACCCTCTGGATGGTC	59	
22	F: GAGGCTGAGATTGCACCACTG	59	765
	R: CCTTACCAGTCCTAACAAGAGGC	57	
23	F: AGTGCTACTGGGCTCAAGTGC	58	511
	R: GGGATAATGATGACACCCACC	57	
24-1	F: ACAGATTATGTTTGGAGGGGC	57	675
	R: TCACATCTTGTATCCCCATCAGTA	57	
24-2	F: TGTGCAACAGCAATGAAATTAAC	56	732
	R: TGCTCTTGGACTGACCAGTC	55	
24-3	F: TAGAGGGAGCAGAAAGGTCAACA	59	771
	R: TCATGCACACAGGTATGGCAA	60	
24-4	F: GTAAAGGAGCAGAAATGTAGTTACA	56	722
	R: AGGTCTACCCTTGTACTCCAGATAT	55	
24-5	F: TTAATTAATAAACGCACAGCCCTA	56	844
	R: GTACCTCTGATGCATTTAGGTGAC	56	
*GLOPH3*			
1	F: TAATTAACTCCCGCGCCGA	60	776
	R: GGGGAGGATCCAGAAAGCA	59	
2	F: TGGGGTTAACTGAGTATTCCTTGG	59	814
	R: TATTGTCCTGTGACCCTGCCA	60	
3	F: GCTACTGAGTCTAGCCAATTTTCAT	56	637
	R: TACCACCACAGCTTAACCTAGCC	58	
4-1	F: GGTCTGGCTAGGCTTAAGGGG	60	613
	R: GAATGGTTCACCCCGAGCA	60	
4-2	F: AAGAGAGTGCGGCAGCTTCTC	60	734
	R: CCCATCCCAAACTGGCTCT	58	
4-3	F: GGCCTTCAACTCACCAAAGGTA	59	679
	R: TACATGCAACATCTGCTAGGACTG	58	
4-4	F: GGCTTGTGACCAGTACCAATCT	57	578
	R: AAACACAAATGACATGCTTGCTC	58	
*ZFR*			
1 and 2	F: TTAAGGAGCCGCGAAGACG	60	664
	R: TTCGTCGCATCGACAGGAT	59	
3	F: GACCTTTTGTGGTCCGTCATT	57	578
	R: GGTATGTCCCAAACTACCAAGG	56	
4	F: GCCAGGATGGTCTTTATCTGCT	58	664
	R: CAGCTTATTTCAGCAGGAATGG	58	
5	F: GGAGAAATTGCTGGCATAAAAT	56	603
	R: CTAAGCCCAAGACTCATAATGAGC	57	
6	F: AAGGTTCTTCAAGGCAGGGAC	58	667
	R: CCCTGAAAATTCTCATGCCAC	58	
7	F: CGGGCGATACAGAGAACCATAG	59	568
	R: GACAGGTCTCCAGTCTTTCCCTC	59	
8	F: GAGGGAAAGACTGGAGACCTGTC	59	893
	R: AGGGTCTTTGTGTGTGCAGATC	58	
9	F: GATGAGGAGGGTGTTGGGTG	59	675
	R: TGCACACCACAGTGGCAATAC	59	
10	F: GAGGTTGTAGTGAGCTGAGTTCAA	56	486
	R: AACCAAACATCCATCTGAGTCTAC	55	
11	F: TACATGTAGATTGTTTTGGGGC	55	492
	R: TTGTTTGAAACCGAGGCACT	57	
12	F: TGGGAAGAAATTTTAGCTAGGCTG	59	516
	R: AAGCTGAGGCAGGAGAATTGCT	60	
13	F: GGTGTACATGCATGCATGCAT	59	569
	R: TGCAAGCAGCTGCAGAATACAT	60	
14	F: GATGGAAATTTTAATGGCACAAA	57	573
	R: TCCTAACAACTGCCTTCTTATGAT	55	
15	F: AATAATACTGGCATGTACGGCAG	57	406
	R: ATGCCAGCATGTTGCCTTCTA	59	
16	F: GGCTCATGTGACACTGATGCTAC	58	366
	R: GCAATATGCAGATCATCATACCC	57	
17	F: CTGAGCTTCCATTGAACGGTG	59	335
	R: CCCAGGATTTTTCATCAGAAAAG	58	
18 and 19	F: GATTACAGACGTGAGCCACTGTG	58	666
	R: TCTAGGGGCTTGCTTACTACAGA	56	
20-1	F: TAGGTTGCATTTGGAGGGAGG	60	774
	R: CAAACTCTGCAGTCTCACGTTACA	58	
20-2	F: CGAGATGGTATCCTTTACCCC	56	570
	R:CACACCAATAAGGAACTGTCACC	57	
20-3	F: CTTGTGTATAAGTGGAAAGGGCA	57	594
	R: GGCCGTGCTTAGACAACAAAC	58	

Forward and reverse primers designed for *CDH6*, *CDH10*, *CDH12*, *PDZD2*, *GLOPH3*, and *ZFR* for mutation screening.

## Results

A large, multigenerational, Chinese family with autosomal dominant high myopia was recruited and characterized (Figure 1). DNA was extracted from 29 blood samples of the family members (10 affected). The average age at diagnosis of myopia in the affected individuals was 6.9 years (range, 4 to 11 years). The average spherical component refractive error for the affected individuals was −11.59±5.26 D (range, −6.5 to −26 D). The mean axial lengths were 29.17±1.50 mm (range, 26.80 mm to 31.42 mm) and 23.59±1.04 mm (range, 22.03 mm to 25.72mm) for highly myopic and non-highly myopic subjects, respectively. Individual III:7 had the highest refractive error of −24.00 D for the right eye and −26.00 D for the left eye (Table 1).

Ophthalmological examination excluded known ocular diseases associated with myopia, including keratoconus, spherophakia, ectopia lentis, retinal dystrophy, and optic atrophy. Males and females in this family were equally affected.

All known syndromic myopia loci were excluded in this family. The LOD scores at θ=0.00 were as follows: D15S117 (Marfan syndrome), −2.43; D1S218 (juvenile glaucoma), −2.5; D12S85 (Stickler syndrome type 1), −6.44; D1S206 (Stickler syndrome type 2), −10.77; and D6S276 (Stickler syndrome type 3), −4.15. Linkage to all of the known loci for non-syndromic autosomal dominant high myopia showed no statistically significant or suggestive evidence of linkage in this family (data not shown). Through subsequent genome-wide screening, a two-point LOD score of 3.02 (θ=0.00) was initially obtained with the microsatellite marker D5S419, suggesting that the causative locus for the family with high myopia was mapped to a region adjacent to D5S419 on chromosome 5. For fine mapping, an additional 13 closely flanking microsatellite markers were tested, and the linkage analysis resulted in a significant LOD score at 5p13.3-p15.1. Seven microsatellite markers displayed positive LOD scores, with D5S502 having the highest LOD score, 3.71 at θ=0.00 (Table 3).

**Table 3:** Two-point linkage analysis between high myopia and markers on chromosome 5p13.3-p15.1.

			**LOD Score at θ=**								
**Marker**	**Marshfield map (cM)**	**Physical Map (Mb)**	**0.00**	**0.01**	**0.05**	**0.10**	**0.20**	**0.30**	**0.40**	**Zmax**	**θmax**
D5S416	28.76	16.74	−2.58	−1.72	−0.72	−0.17	0.27	0.33	0.21	0.33	0.30
D5S486	31.78	17.20	−3.60	−1.91	−0.61	0.00	0.44	0.46	0.28	0.46	0.30
D5S2096	33.04	17.47	−1.33	−1.13	−0.59	−0.22	0.10	0.14	0.05	0.14	0.30
D5S2074	36.25	21.15	2.00	2.02	2.01	1.92	1.57	1.07	0.46	2.02	0.01
D5S813	37.32	23.48	2.86	2.81	2.60	2.35	1.83	1.22	0.53	2.86	0.00
D5S502	39.46	25.57	3.71	3.67	3.48	3.20	2.53	1.73	0.80	3.71	0.00
D5S419	39.99	26.54	3.02	2.99	2.84	2.61	2.05	1.37	0.60	3.02	0.00
D5S385	39.99	27.34	1.92	1.87	1.68	1.45	0.99	0.52	0.12	1.92	0.00
D5S2061	41.06	29.86	1.69	1.66	1.52	1.34	0.99	0.58	0.21	1.69	0.00
D5S819	41.06	30.75	2.23	2.22	2.05	1.83	1.36	0.86	0.37	2.23	0.00
D5S1986	44.73	31.61	−5.03	−1.53	−0.14	0.36	0.61	0.50	0.25	0.61	0.20
D5S1470	45.34	32.37	−1.41	−0.83	0.27	0.66	0.80	0.62	0.29	0.80	0.20
D5S674	47.09	33.42	−3.43	−1.77	−0.99	−0.60	−0.18	0.01	0.07	0.07	0.40
D5S426	51.99	34.64	−6.00	−5.06	−3.16	−1.99	−0.82	−0.28	−0.04	−0.04	0.40

Fine mapping demonstrated strong evidence of a suggestive linkage for a locus between D5S2096 and D5S1986 on chromosome 5p13.3-p15.1. LOD scores were generated using a dominant mode of inheritance with 90% penetrance and with a disease-gene allele frequency of 0.01.

Haplotype analysis of the affected individuals revealed recombination events that narrowed the region containing the gene, as shown in Figure 1. Through haplotype analysis, it was discovered that in addition to the ten affected individuals, two unaffected siblings (III:15 and III:19) also inherited the putative disease allele. At the time of examination, III:15 and III:19 were 41 and 58 years of age, respectively, and it was not likely that they would develop high myopia. Interestingly, III:15 had only read for 5 years in primary school and III:19 had never attended school: both of them had spent less time reading.

The critical region was found to be between the markers D5S2096 and D5S1986. A telomeric recombinant event occurred between markers D5S2096 and D5S2074 in the affected individual III:17, which defined the distal limit of the region to marker D5S2096. Affected individual IV:9 displayed evidence of a centromeric recombinant event between markers D5S1986 and D5S1470. Another centromeric recombination event was observed between markers D5S819 and D5S1986 in two affected individuals, III:7 and III:11. These defined the proximal limit of the region to marker D5S1986. Ultimately, we mapped high myopia to a locus on chromosome 5p13.3-p15.1, covering an approximately 11.69 cM (14.14 Mb) region between D5S2096 and D5S1986.

Within the linkage region, the six genes cadherin 6, type II (*CDH6*), cadherin 10, type II (*CDH10*), cadherin 12, type II (*CDH12*), PDZ domain-containing protein 2 (*PDZD2*), Golgi phosphoprotein 3 (*GOLPH3*), zinc finger RNA binding protein (*ZFR*) were selected as candidate genes on the basis of their function: cell adhesion, intracellular signal transduction, protein trafficking, and DNA/RNA binding activities, which we thought were the functions most likely to be associated with myopia. A description of these genes was provided in Table 4. However, mutation analysis did not reveal any disease-causing mutation.

**Table 4:** Description of candidate genes located in the linkage interval of 5p13.3-p15.1.

**Symbol**	**Full name**	**Function**	**Cellular component**
*CDH6*	Cadherin 6, type II (K- cadherin)	Cell adhesion, development, neuron adhesion	Cytoplasm
*CDH10*	Cadherin 10, type II (T2-cadherin)	Cell adhesion, development, neuron adhesion	Cytoplasm
*CDH12*	Cadherin 12, type II (Br- cadherin)	Cell adhesion, development, neuron adhesion	Cytoplasm
*PDZD2*	PDZ domain-containing protein 2	Intracellular signal transduction, Control plasticity of synapses	Cytoplasm
*GOLPH3*	Golgi phosphoprotein 3	Regulate protein trafficking	Cytoplasm
*ZFR*	Zinc finger RNA binding protein	DNA and RNA binding, DNA repairing activity	Nucleus; Cytoplasm

## Discussion

In this study, a locus for autosomal dominant high myopia in a large Chinese family was identified. Genome screening and linkage analysis located a critical region for high myopia on chromosome 5p13.3-p15.1 between D5S2096 and D5S1986, within an 11.69 cM interval. Linkage to the candidate gene regions for the Stickler syndromes, Marfan syndrome, and juvenile glaucoma was excluded, ensuring that this family did not exhibit a mild phenotypic expression of these conditions. Similarly, linkage was excluded from known autosomal myopia loci. This study has provided additional evidence for the genetic heterogeneity of autosomal dominant high myopia.

Myopia is thought to be multifactorial, caused by a variety of environmental and genetic factors as well as their interactions. Compared to the remarkable progress in identifying the genes for retinal degeneration, genes causing non-syndromic myopia (common or high) have proven difficult to identify. The likely explanation for this difficulty is that the gene-environment interplay affects even Mendelian patterns of myopia [1,12,13]. The underlying pattern of genetic and/or environmental factors in myopic subjects is highly variable and incomplete penetrance is common in high myopia, as reported in other high myopia families [26,32]. It was noted that all patients with high myopia in this family carried the putative disease haplotype; but two individuals, III:15 and III:19, both of whom inherited the putative disease allele from their mother, did not have high myopia. At the time of examination, III:15 and III:19 were 41 and 58 years of age, respectively, and it was not likely that they would develop high myopia. Interestingly, III:15 had only read for 5 years in primary school and III:19 had never entered school, so these two siblings had spent less time reading. These suggested that the variability in the phenotype might be mostly attributable to the interplay of genetic and environmental factors, leading to incomplete penetrance of the disease.

Six candidate genes *CDH6*, *CDH10*, *CDH12*, *PDZD2*, *GOLPH3*, and *ZFR* at this high myopia locus were selected on the basis of their function to screen for gene mutations by re-sequencing. The classical cadherins mediate homophilic cell–cell adhesion and are key regulators of many morphogenetic processes [33].

Loss-of-function studies demonstrate that the classical cadherins play a crucial role in vertebrate retinogenesis. They have multiple morphoregulatory functions in retinal proliferation, migration, differentiation, and layer formation, as well as axonal outgrowth, pathfinding, target recognition, and synaptogenesis [34,35]. *CDH6* regulates the differentiation of retinal ganglion cells, amacrine cells, and photoreceptors in Zebrafish [36]. *CDH10* and *CDH12* were detected in the mouse eye during the first postnatal week when several developmental processes, such as cell migration and formation of synaptic connections, occur simultaneously [37]. *PDZD2* is a ubiquitously expressed multi-PDZ-domain protein [38]. PDZ domain scaffolds have been shown by genetic, electrophysiological, and morphological studies to be essential for controlling the structure, strength, and plasticity of synapses, which may play a role in the process of vision formation [39]. *GOLPH3* is a peripheral membrane protein of the Golgi stack. It is required for trafficking from the Golgi to the plasma membrane and for the normal extended Golgi ribbon. Depletion of *GOLPH3* alters the Golgi ribbon, changing its normal appearance of extending partially around the nucleus, to condensing at one end of the nucleus [40]. *ZFR* contains three widely spaced zinc finger domains. Zinc finger proteins with a similar pattern of zinc finger motifs are known to bind RNA, DNA, and DNA/RNA hybrids [41]. *ZFR* can be involved in DNA repair and chromosome organization [42]. Analyses of *ZFR* knockout mice indicate that *ZFR* is essential for at least some developmental pathways, as embryonic death occurs at 8–9 days gestation in these mice. In homozygotes, genetic ablation of *ZFR* causes increased embryonic cell death and/or decreased cell proliferation rates [43]. In the current study, PCR and sequencing primers were synthesized for the exons and peripheral intron regions of these candidate genes, and direct sequencing analysis was performed. However, no disease mutation was identified.

**Figure 2:**
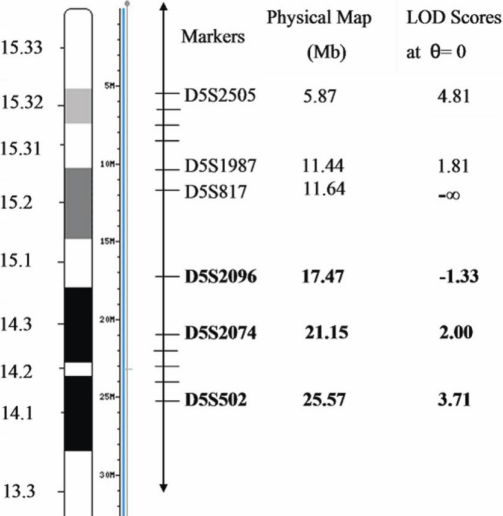
The position relationship between the locus and MYP16 (5p15.33–15.2) on the short arm of chromosome 5. The upper three markers were identified in MYP16 locus 5p15.33–15.2: D5S2505 was the marker with the peak two-point LOD score 4.81. D5S1987 was the marker close to centromeric with positive LOD score while D5S817 was the next marker displaying negative LOD score. The lower three markers were identified in the present study (bold text): D5S502 was the marker with the highest two-point LOD score 3.71. D5S2074 was the first telomeric marker having positive LOD score. The physical distance between D5S2505 and D5S502 was approximately 19.7Mb and the physical distance between D5S1987 and D5S2074 was 9.71Mb. In addition, the physical distance of the nearest two markers displaying negative linkage to high myopia in these two studies (D5S817 and D5S2096) was 5.83Mb.

A previous study revealed an autosomal dominant high myopia locus mapped to chromosome 5p15.33-p15.2 with an interval of 17.45 cM between D5S1970 and D5S1987 in three Chinese pedigrees originating from Hong Kong (HK) [27]. In our study, the locus for high myopia of the pedigree was mapped to the critical region between D5S2096 and D5S1986 on chromosome 5p13.3-p15.1. The physical distance of the two markers yielding the peak two-point LOD score (D5S2505 in HK families and D5S502 in Zhejiang family) is approximately 19.7 Mb. The physical distance of the nearest two markers displaying a strong linkage to high myopia in these two studies (D5S1987 and D5S2074) was 9.71 Mb. However, the linkage regions with high myopia on the short arm of chromosome 5 identified in these two studies did not show any overlap (Figure 2). The fact that the two causative loci identified in these Chinese families with inherited high myopia did not overlap, but were adjacent, suggested that there may be disease gene(s) for high myopia on the short arm of chromosome 5 in the Chinese population. Although no mutation has yet been identified for the putative candidate genes, a more refined mutation screen is needed to identify the causative gene(s).

In summary, we have mapped a genetic locus for autosomal dominant high myopia in a large Chinese family. Myopia is the most common eye disease. Identification of the mutant gene(s) for myopia potentially would advance the understanding of the causes of this common eye disorder, and may thus lead to methods for preventing or slowing its progression.

## References

[1] Feldkämper M Schaeffel F Interactions of genes and environment in myopia. Dev Ophthalmol 2003 37 34 49 12876828 10.1159/000072037

[2] Yamada M Hiratsuka Y Roberts CB Pezzullo ML Yates K Takano S Miyake K Taylor HR Prevalence of visual impairment in the adult Japanese population by cause and severity and future projections. Ophthalmic Epidemiol 2010 17 50 7 20100100 10.3109/09286580903450346

[3] Wong TY Foster PJ Hee J Ng TP Tielsch JM Chew SJ Johnson GJ Seah SL Prevalence and Risk Factors for Refractive Errors in Adult Chinese in Singapore. Invest Ophthalmol Vis Sci 2000 41 2486 94 10937558

[4] He M Huang W Zheng Y Huang L Ellwein LB Refractive error and visual impairment in school children in rural southern China. Ophthalmology 2007 114 374 82 17123622 10.1016/j.ophtha.2006.08.020

[5] Fan DS Lam DS Lam RF Lau JT Chong KS Cheung EY Lai RY Chew SJ Prevalence, incidence, and progression of myopia of school children in Hong Kong. Invest Ophthalmol Vis Sci 2004 45 1071 5 15037570 10.1167/iovs.03-1151

[6] Wang Q Klein BE Klein R Moss SE Refractive status in the Beaver Dam Eye Study. Invest Ophthalmol Vis Sci 1994 35 4344 7 8002254

[7] Wensor M McCarty CA Taylor HR Prevalence and risk factors of myopia in Victoria, Australia. Arch Ophthalmol 1999 117 658 63 10326965 10.1001/archopht.117.5.658

[8] Katz J Tielsch JM Sommer A Prevalence and risk factors for refractive errors in an adult inner city population. Invest Ophthalmol Vis Sci 1997 38 334 40 9040465

[9] Hochman MA Seery CM Zarbin MA Pathophysiology and management of subretinal hemorrhage. Surv Ophthalmol 1997 42 195 213 9406367 10.1016/s0039-6257(97)00089-1

[10] Banker AS Freeman WR Retinal detachment. Ophthalmol Clin North Am 2001 14 695 704 11787748 10.1016/s0896-1549(05)70268-6

[11] Saw SM Gazzard G Shih-Yen EC Chau WH Myopia and associated pathological complications. Ophthalmic Physiol Opt 2005 25 381 91 16101943 10.1111/j.1475-1313.2005.00298.x

[12] Saw SM A synopsis of the prevalence rates and environmental risk factors for myopia. Clin Exp Optom 2003 86 289 94 14558850 10.1111/j.1444-0938.2003.tb03124.x

[13] Schaeffel F Simon P Feldkaemper M Ohngemach S Williams RW Molecular biology of myopia. Clin Exp Optom 2003 86 295 307 14558851 10.1111/j.1444-0938.2003.tb03125.x

[14] Teikari JM O’Donnell J Kaprio J Kosenvuo M Impact of heredity in myopia. Hum Hered 1991 41 151 6 1937488 10.1159/000153994

[15] Teikari JM Kaprio J Koskenvuo M O’Donnell J Heritability of defects of far vision in young adults: a twin study. Scand J Soc Med 1992 20 73 8 1496333

[16] Liang CL Yen E Su JY Liu C Chang TY Park N Wu MJ Lee S Flynn JT Juo SH Impact of family history of high myopia on level and onset of myopia. Invest Ophthalmol Vis Sci 2004 45 3446 52 15452048 10.1167/iovs.03-1058

[17] Schwartz M Haim M Skarsholm D X-linked myopia: Bornholm eye disease. Linkage to DNA markers on the distal part of Xq. Clin Genet 1990 38 281 6 1980096

[18] Zhang Q Guo X Xiao X Jia X Li S Hejtmancik JF Novel locus for X-linked recessive high myopia maps to Xq23–q25 but outside MYP1. J Med Genet 2006 43 e20 16648373 10.1136/jmg.2005.037853 PMC2564525

[19] Yang Z Xiao XS Li SQ Zhang QJ Clinical and linkage study on a consanguineous Chinese family with autosomal recessive high myopia. Mol Vis 2009 15 312 8 19204786 PMC2635848

[20] Young TL Ronan SM Drahozal LA Wildenberg SC Alvear AB Oetting WS Atwood LD Wilkin DJ King RA Evidence that a locus for familial high myopia maps to chromosome 18p. Am J Hum Genet 1998 63 109 19 9634508 10.1086/301907 PMC1377231

[21] Young TL Ronan SM Alvear AB Wildenberg SC Oetting WS Atwood LD Wilkin DJ King RA A second locus for familial high myopia maps to chromosome 12q. Am J Hum Genet 1998 63 1419 24 9792869 10.1086/302111 PMC1377552

[22] Naiglin L Gazagne C Dallongeville F Thalamas C Idder A Rascol O Malecaze F Calvas P A genome wide scan for familial high myopia suggests a novel locus on chromosome 7q36. J Med Genet 2002 39 118 24 11836361 10.1136/jmg.39.2.118 PMC1735027

[23] Paluru P Ronan SM Heon E Devoto M Wildenberg SC Scavello G Holleschau A Makitie O Cole WG King RA Young TL New locus for autosomal dominant high myopia maps to the long arm of chromosome 17. Invest Ophthalmol Vis Sci 2003 44 1830 6 12714612 10.1167/iovs.02-0697

[24] Zhang Q Guo X Xiao X Jia X Li S Hejtmancik JF A new locus for autosomal dominant high myopia maps to 4q22-q27 between D4S1578 and D4S1612. Mol Vis 2005 11 554 60 16052171

[25] Paluru PC Nallasamy S Devoto M Rappaport EF Young TL Identification of a novel locus on 2q for autosomal dominant high-grade myopia. Invest Ophthalmol Vis Sci 2005 46 2300 7 15980214 10.1167/iovs.04-1423

[26] Nallasamy S Paluru P Devoto M Wasserman NF Zhou J Young TL Genetic linkage of high-grade myopia in a Hutterite population from South Dakota. Mol Vis 2007 13 229 36 17327828 PMC2633468

[27] Lam CY Tam PO Fan DS Fan BJ Wang DY Lee CW Pang CP Lam DS Genome-wide Scan Maps a Novel High Myopia Locus to 5p15. Invest Ophthalmol Vis Sci 2008 49 3768 78 18421076 10.1167/iovs.07-1126

[28] Stambolian D Ibay G Reider L Dana D Moy C Schlifka M Holmes T Ciner E Bailey-Wilson JE Genomewide linkage scan for myopia susceptibility loci among Ashkenazi Jewish families shows evidence of linkage on chromosome 22q12. Am J Hum Genet 2004 75 448 59 15273935 10.1086/423789 PMC1182023

[29] Hammond CJ Andrew T Mak YT Spector TD A susceptibility locus for myopia in the normal population is linked to the PAX6 gene region on chromosome 11: a genomewide scan of dizygotic twins. Am J Hum Genet 2004 75 294 304 15307048 10.1086/423148 PMC1216063

[30] Wojciechowski R Moy C Ciner E Ibay G Reider L BaileyWilson JE, Stambolian D. Genomewide scan in Ashkenazi Jewish families demonstrates evidence of linkage of ocular refraction to a QTL on chromosome 1p36. Hum Genet 2006 119 389 99 16501916 10.1007/s00439-006-0153-x PMC3123998

[31] Ciner E Wojciechowski R Ibay G Bailey-Wilson JE Stambolian D Genomewide scan of ocular refraction in African-American families shows significant linkage to chromosome 7p15. Genet Epidemiol 2008 32 454 63 18293391 10.1002/gepi.20318 PMC3097031

[32] Farbrother JE Kirov G Owen MJ Guggenheim JA Family aggregation of high myopia: estimation of the sibling recurrence risk ratio. Invest Ophthalmol Vis Sci 2004 45 2873 8 15326097 10.1167/iovs.03-1155

[33] Takeichi M Morphogenetic roles of classic cadherins. Curr Opin Cell Biol 1995 7 619 27 8573335 10.1016/0955-0674(95)80102-2

[34] Hirano S Suzuki ST Redies C The cadherin superfamily in neural development: diversity, function and interaction with other molecules. Front Biosci 2003 8 d306 55 12456358 10.2741/972

[35] Takeichi M The cadherin superfamily in neuronal connections and interactions. Nat Rev Neurosci 2007 8 11 20 17133224 10.1038/nrn2043

[36] Liu Q Londraville R Marrs JA Wilson AL Mbimba T Murakami T Kubota F Zheng WP Fatkins DG Cadherin-6 Function in Zebrafish Retinal Development. Dev Neurobiol 2008 68 1107 22 18506771 10.1002/dneu.20646 PMC2562688

[37] Faulkner-Jones BE Godinho LN Tan SS Multiple Cadherin mRNA Expression and Developmental Regulation of a Novel Cadherin in the Developing Mouse Eye. Exp Neurol 1999 156 316 25 10328938 10.1006/exnr.1999.7026

[38] Yeung ML Tam TS Tsang AC Yao KM Proteolytic cleavage of PDZD2 generates a secreted peptide containing two PDZ domains. EMBO Rep 2003 4 412 8 12671685 10.1038/sj.embor.embor804 PMC1319160

[39] Kim E Sheng M PDZ domain proteins of synapses. Nat Rev Neurosci 2004 5 771 81 15378037 10.1038/nrn1517

[40] Dippold HC Ng MM Farber-Katz SE Lee SK Kerr ML Peterman MC Sim R Wiharto PA Galbraith KA Madhavarapu S Fuchs GJ Meerloo T Farquhar MG Zhou H Field SJ GOLPH3 bridges phosphatidylinositol-4-phosphate and actomyosin to stretch and shape the Golgi to promote budding. Cell 2009 139 337 51 19837035 10.1016/j.cell.2009.07.052 PMC2779841

[41] Finerty PJ Jr Bass BL A Xenopus zinc finger protein that specifically binds dsRNA and RNA–DNA hybrids. J Mol Biol 1997 271 195 208 9268652 10.1006/jmbi.1997.1177

[42] Meagher MJ Schumacher JM Lee K Holdcraft RW Edelhoff S Disteche C Braun RE Identification of ZFR, an ancient and highly conserved murine chromosome-associated zinc finger protein. Gene 1999 228 197 211 10072773 10.1016/s0378-1119(98)00615-5

[43] Meagher MJ Braun RE Requirement for the murine zinc finger protein ZFR in perigastrulation growth and survival. Mol Cell Biol 2001 21 2880 90 11283266 10.1128/MCB.21.8.2880-2890.2001 PMC86917

